# Structured multi-criteria model of self-managed motivation in organizations based on happiness at work: pandemic related study

**DOI:** 10.1038/s41598-023-43626-5

**Published:** 2023-10-02

**Authors:** Joanna Nieżurawska, Radosław A. Kycia, Iveta Ludviga, Agnieszka Niemczynowicz

**Affiliations:** 1 Faculty of Finance and Management, WSB Merito University in Toruń, Toruń, Poland; 2 Department of Mathematics and Statistics, Masaryk University, Brno, Czech Republic; 3 Faculty of Computer Science and Telecommunications, Cracow University of Technology, Kraków, Poland; 4 Riseba University of Applied Sciences, Riga, Latvia; 5 Faculty of Mathematics and Computer Science, University of Warmia and Mazury in Olsztyn, Olsztyn, Poland

**Keywords:** Human behaviour, Scientific data, Statistics

## Abstract

This study proposes the Structured Multi-criteria Model of Self-Managed Motivation in Organizations based on happiness at work. Employees need to be motivated in order to be efficient at doing a particular task at the workplace. As young people born between 1995 and 2004, called Generation Z, enter the labour market, it is essential to consider how employees’ motivation might be affected. In the article a quantitative approach was used to collect new data from 200 Polish respondents completing an online survey. The research was conducted before and during the pandemic time. We report and analyze the survey results conducted in Poland among the representatives of Generation Z, who had been employed for at least 6 months. We developed and validated a new approach to motivation using methodologies called Factor Analysis. Based on empirical verification, we found a new tool that connects employee motivation and selected areas of the Hygge concept called *Hygge Star Model*, which has the same semantics before and during Covid-19 pandemic.

## Introduction

The objective of this paper is to present a Structured Multi-criteria Model of Self-Managed Motivation in Organizations based on happiness at work-validated on surviving interminable disruptions e.g., the corona virus COVID-19 pandemic. It is known that employees’ productivity is affected by the policy chosen to motivate them^1,3,59^. High motivation to work is synonymous with achieving a harmonious and happy life in all areas of functioning. Employee workplace motivation is essential to achieve high levels of customer satisfaction. According from Sørensen and Sorensen companies gain from highly motivated employees working towards common goals^61^. Nevertheless, surviving interminable disruptions e.g., Covid-19 profoundly affects almost all aspects of economic and social life globally, as well as the organization area. Measures taken to protect public health have threatened the global economy, necessitating economic stimulus in most countries, and reconfiguring the role of business and work value^6,19^.

We focus on young representatives of Generation Z, also called GenZ. Generation Z are people born between 1995–2004 and we know that their entrance into the labor market as well as their number and strength will be bigger. We know that there is a need for a new way of motivating^45,51,66^. According to Schullery^60^ each generational group differs in their values and characteristics that exert a direct impact on attitudes and behaviours. That is why it’s employers’ job to detect and understand the generational difference, which may predict motivation to perform the job^66^. Moreover, organisations constantly need to work on changing organizational practices to adapt to the diverse nature of the workforce consisting of people from different generations. For service organisations, in particular, creating a quality internal working environment is crucial in order to drive employee satisfaction^57^. We believe, that it is necessary to understand generational differences in the workplace in order to create a positive work culture and improve employee engagement and motivation^39^. There is a gap in the following respect, which has become a premise for undertaking the research presented in this article.

### The problem and the approach

According to Churchill^8^, a measure is valid when the differences in observed scores reflect true differences on the characteristic. We also realized that a measure is reliable to the extent that independent but comparable measures of the same trait or construct of a given object agree. We found that reliability depends on how much of the variation in scores is attributable to random or chance errors^8^.

We introduce the research problem in the form of the following questions:

1. Can Hygge concept be used to motivate young people, born between 1995 and 2004, called Generation Z?
2. How did Hygge Star Model change during the pandemic time?
3. Can we define the future changes in the Hygge Model on surviving interminable disruptions?

We formulated a hypothesis:

1. The Hygge Star Model dimensions to employees’ overall work motivation are valid but not invariant across surviving interminable disruptions (e.g., the corona virus COVID-19 pandemic).

The paper is organized as follows. The next section discusses the theoretical models of motivations in the workplace. We focus on self-determination theory, health and well-being as the background. Next, we present the hygge concept and generational differences in the workplace. After that, we demonstrate the empirical part describing methodology and data analysis using factor analysis. The paper ends with a discussion, strengths, limitations, and conclusions.

## Theory-the body of conceptual knowledge

### Self-determination theory

Self-determination theory (SDT) defines three basic needs as essential to psychological health and well-being: autonomy, competence, and relatedness. Self-determination theory realizes a multidimensional conceptualisation of motivation comprising autonomous and controlled forms. Autonomous motivation relates positively to individuals’ optimal functioning (e.g., well-being, performance), controlled motivation is less beneficial. It is possible to use self-determination theory in the field of organizational behavior^23^. SDT proposes a multidimensional view on motivation and specifies how these different types of motivation can be promoted or discouraged. Three major categories of motivation are discerned^23^. First, amotivation is defined as the absence of motivation towards an activity. Second, intrinsic motivation is defined as doing an activity for its own sake, that is, because it is interesting and enjoyable in itself. Third, extrinsic motivation refers to engaging in the activity for instrumental reasons, such as receiving rewards and approval, avoiding punishments or criticism, boosting one’s self-esteem, or reaching a personally valued goal. SDT characterizes different subtypes of extrinsic motivation, which vary in their internalization. Internalization is understood as taking in a value-driven or goal-driven activity that was initially regulated by external factors, such as rewards or punishments, so that it becomes internally regulated^16^. A number of researchers have offered external and introjected regulations into a controlled motivation composite score and combined identified and intrinsic motivation into an autonomous motivation composite score^67^. When the regulation of a behavior has been well internalized, people identify with its personal value for themselves and thus perform the behavior volitionally because of its importance for their own lives and self-selected goals. This is referred to as identified regulation^67^. In opposition to controlled motivation and amotivation, autonomous motivation has been found to yield the most desirable behavioral, attitudinal, and affective outcomes^16^.

### Conceptualizations of health and well-being

Well-being in the workplace is an important issue that should occupy a much more prominent niche in mainstream organizational research for some reasons^15^. First, an individual’s experiences at work, be they emotional or social in nature, obviously affect the person while working. Furthermore, these experiences also ’spill over’ into non-work domains. Workers spend about one-third of their time at work, and don’t necessarily leave the job behind when they leave the work site^11^. Second, well-being can potentially affect both workers and organizations in negative ways. Workers with poor well-being may be less productive, make lower quality decisions, be more prone to be absent from work, and make consistently diminishing overall contributions to the organizations^53^. Factors that influence employee health and well-being can have a significant impact on the financial health and profitability of an organization^12^. Currently the health and well-being of workers is becoming an increasingly important issue. Corporations have long been involved in health issues in terms of occupational health and safety, providing disability and insurance packages and employee assistance programs^10^. A majority of employees’ compensation packages include healthcare coverage health and well-being constructs, and their associated research is quite evident, given the implications of workplace dimensions that interact with individual level factors affecting workers’ overall experiences of work and life^15^. Finally, the role of interventions is highlighted showing their potential impact on antecedent factors, actual well-being, and the consequential factors. For example, many interventions targeted at the organizational and individual levels have been implemented in an attempt to improve the safety and working conditions in the workplace, alleviate or lessen potential occupational stressors, or improve the individual’s coping mechanisms with these stressors. This, in turn, should correlate to increased employee well-being and health with concomitant improvements in individual and organizational consequences^17^.

### Conceptualizations of hygge concept

Researchers proposed that the employees’ satisfaction depends on achievements. They suggest that higher achievements determine higher satisfaction. There are two variables, which influence achievements: ability and role perception. It is also observed that employers and companies are looking for new ways to stimulate their employees towards being more productive and happier at the same time^2^. An example of happiness at work could be the introduction of hygge to the workplace. Jeppe Trolle Linnet^36^ described hygge as a social interaction style connected with cultural values. Black and Bodkaer^4^ think likewise, seeing hygge as a particular type of social interaction which should be safe and made between people with whom spending time is enjoyable^36,38^. According to Linnet^36^, hygge works well in private life as well as in commercial setting. In the latter one, hygge can be applied in most of the human resources management areas, but also in the way the enterprise operates in the business environment. The main areas where hygge can be implemented include motivating the employees, work organization, but also issues related to the structures within the organization or business activities^43^. Hygge is a concept of happiness. It can be reflected in theories of needs in the sense that desirable happiness at work can come from meeting various needs. In HRM management Hygge in the commercial setting is characterized by the presence of greenery, which aim is to relax and calm down the employees during their work^44^. Another need connected with work is egalitarianism and transparency at the workplace, which translates to equality of all employees and transparency of motivating and remuneration policies. Fair play and lack of aggressive behaviors is yet another need accounted for in the pyramid of employees’ needs carried out according to the concept of hygge^44^. The organizational culture of a company which operates according to the concept of hygge is based on mutual trust, teamwork, transparency of actions and decisions, as well as chill, liberty, and spontaneity^44^. Hygge also means appropriate working conditions which include a place where a team can eat lunch or have a coffee break. It is important to create a cosy office space, equipped with not only the necessary supplies, but also with abundant greenery, good lighting, and surrounding oneself with personal items-such as a favourite mug on the desk. The need is also visible in creating a cosy and modern office space which is dominated by the colors of nature: earth (brown and green) and the sky (blue)^44^. Hygge at the workplace is a style aimed at teamwork, which includes brainstorming, shared problem-solving, conversations, as well as project meetings. According to hygge, the organizational structure of an enterprise should be flat, which fosters transparency and better communication. According to the concept of hygge, motivating the employees is achieved by increasing their engagement thanks to clear and precise goals, proper employee evaluation, and consistent feedback from the employer/manager^44^. The first validation questionnaire of happiness at work according to Linnet research^36^ was valid in Poland after testing 1100 people working in companies^44^.

## Generational blend in the workplace

According to the Pew Research Center (2015), while setting the age boundaries of generations is a necessary step for generational analysis, the lines that define the generations should be thought of as guidelines, rather than hard and fast distinctions^50^. The concept of ’generation’ defined as groups of people born in a similar time, is utilised as an approach for grouping age cohorts, as well as for the analysis for tracking people on behaviors and characteristics^39^. Young people from Generation Z, also called GenZ, are people born between 1995-2004, Generation Y between 1977-1994, Generation X between 1965-1979, and Baby boomers between 1946-1964^39,45^. Gen Z also known as: Generation C, Post-Millennials, the iGeneration, Founders, Plurals, Homeland^44^. Employers hire representatives of generation Z with little concern because their experience shows that Zs are demanding, sophisticated and unprepared for hard work. They know what they want, and they often think of themselves as exceptional individuals. The fact that they are perceived as mobile people, freely using the resources of the Internet, and creatively using its potential, speaks in favour of Zs. There is a noticeable fluctuation among young employees, because Zs change jobs very often^44^. For each generation, we can talk about slightly different life priorities and adopting different attitudes towards professional work. Although the generational groups emphasize their individuality, none of them can exist in isolation from the rest. Therefore, the characteristics of Zs will be more readable if we first build the context and present it against older generations. Each generation has strong cultural connections, which is why it is affected by socio-cultural mechanisms^34,42,43^. They lead an active professional and private life in social media, carefully building social media profiles and ‘pampering’ their internet image (Snapchat, Instagram, WhatsApp, Facebook, TikTok). They are global in social and technological terms, they lead a lush virtual life, modern technologies are an inseparable element of their daily functioning. They are always online and in constant contact^43,44^ According to Mahmoud et all^39^ Generation Z is more sensitive to amotivation than Generation X and Generation Y. Extrinsic regulation-material is a valid source of overall work motivation for Generation Z only. Only Generation X values extrinsic regulation-social as a source of employees’ overall motivation. So is introjected regulation by Generation Y. Unlike Generation Z, both Generation X and Generation Y employees value identified regulation as a source of overall work motivation. Finally, intrinsic motivation contributes more to Generation Z employees’ overall work motivation than it does for Generation X and Generation Y^34,44^. The unrealistic expectations of young workers are due to their lifestyle, with constant access to online resources, where they find their authority^44^. There is a very high turnover among young employees, as Z change jobs very often. According to research-this generation has a lower correlation between commitment and loyalty than previous generations. Even if Z are committed to their tasks, they are willing to change jobs in order to search for better employment conditions (no loyalty to the employer, they change jobs without sentiment)^43,45^. They value their independence and openness to change because life as they know it is fast-paced; new information is constantly emerging and much of it becomes outdated due to the influx of new data. Generation Z feels safer and more secure in the virtual world than in the real world, so they make new acquaintances-friendships and partnerships-in virtual communities. This attitude makes it harder for them to work as part of a team, while at the same time making them leaders in online collaboration. Such conclusions are also reached by Ludviga and Sluka^37^, Duarte^20^, Nieżurawska and Galaś^44^.

## Methods

### Questionnaire

To examine the motivation system of young people employees in the workplace, an online survey was used. The techniques and technologies used in survey research advanced dramatically during the twentieth century, from systematic sampling methods to improved questionnaire design and compared data analysis. Technology, in particular, has revolutionized how surveys are administered over the last 25 years, with the introduction of the first e-mail survey in the 1980s and the first web-based surveys in the 1990s^22,58^. Although the characteristics of online surveys have been extensively described in the literature^22^, online surveys have a number of practical advantages and disadvantages. The online survey was divided into eight sections, but in this study we analyse only one of them (as shown in Supplementary information), but included two periods of time, before and during Covid-19. The section dealt with a specific piece of information that was required to precisely investigate our research problem.

Only one part of the questionnaire was examined in the current study: this concerning employee motivation systems from the standpoint of their expectations (what are their expectations with regard to the motivation systems). The data were collected in two periods: before Covid-19, it means from October 1st to November 1st, 2020 (the first stage of the research), and during the Covid-19, from May 1st to December 1st, 2021 (the second stage of the research).

This study took into account the following aspects of an online survey: *Q3* (modern systems and concepts of remuneration and motivation, 8 items): The researchers will investigate the significance of a new approach to motivation in this section.

A five-point Likers scale (1 → unimportant, 2 → not so important, 3 → moderately important, 4 → important, 5 → very important) was used for each items. A reliability analysis is conducted from the two constructs used in this study.

From the two constructs used in this study, a reliability analysis is performed. The primary goal of this stage of the research was to investigate the appropriateness of the items and the internal structure of the constructs that the instrument measures.

To test the reliability of the preliminary questionnaire set, reliability on pilot items was performed. The consistency, stability, and dependability of the scores are all factors in an instrument’s or questionnaire’s reliability^9^.

The internal consistency Cronbach’s alpha^14^ and McDonald’s omega^40,56^ were determined to assess reliability. Internal consistency is excellent if the coefficient value is greater than 0.9, the value 0.8 is standard threshold for empirical studies, and acceptable if the coefficient value is greater than 0.7^47,56,69^. Internal consistency indicates that the survey items tend to cluster together. In other words, a participant who responds positively to one survey item is more likely to respond positively to other survey items.

### Study design

According to Hofstede’s studies^27^ the participating samples vary in terms of collectivism and power distance, though some of these values are changing in the younger generations. To test for convergent and discriminant validity, we followed the hygge concept, which was first validated in 2018 in Poland in the group of 1100 people from different generations. The result is that the biggest correlation is between Generation Z and the Hygge concept in the motivation aspects^43^

The study was reviewed and approved by the Institutional Review Board and Ethics Commitee of WSB University in Toruń (Poland). Various Internet Platforms used the quatrix^55^ to send the proprietary questionnaire. Prizes were distributed among survey participants to encourage potential respondents to complete the questionnaire. The online questionnaire was available in two periods: before Covid-19, that is from October 1st to November 1st, 2020 (the first stage of the research), and during the Covid-19, from May 1st to December 1st, 2021 (the second stage of the research).

### Factor analysis

In order to establish the validity of the construct, a preliminary study was conducted to determine the unidimensionality of factors with the use of *Exploratory Factor Analysis* (EFA) through a maximum likelihood extraction method^63^. A Equimax rotation has been applied to factorial analysis to identify each variable with a single factor.

Factor analysis is useful in many research areas, e.g. psychology and the social sciences^25^. It helps to get an understanding of the general picture behind the motivation of Generation Z. EFA is a data reduction method applied to a large set of items to identify an underlying factor structure^63^. We applied *Principal Component Analysis* (PCA) extraction with Equimax rotation, which allows for the interpretation of the factor structure. This method allows each item to be loaded highly on one factor and minimize loadings on the remaining factors.

Firstly, we examined the factorability of the part statements related to modern systems and concepts of remuneration and motivation at the workplace. In order to identify the optimum number of factors, we used *the scree test criterion* (Catell’s method,^7^ or, in the case when this criterion did not work well, *Kaiser criterion*^30,31^. Identification of the factors indicated strong interrelated items of the questionnaire. The Kaiser method assumes the extraction of the factors for which the eigenvalues are greater than one. It suggests that the corresponding factor explains more variance than a single variable^30,31^. The scree test present visual interpretation of the eigenvalues curve of factors. The graph represents the eigenvalues of factors (Y axis) to the corresponding factors (X axis). In this test is that a few major factors account for the most variance. The first, in the graph, we should to find the end of steep “cliff”, and count these factors which are above this point. This way we determine the number of extracted factors. Followed the “cliff” by a shallow “scree”^7^. Factorial loads higher than 0.5 were considered as acceptable^63^.

The results obtained during the factor analysis were archived and statistically analyzed using Python library FactorAnalyzer^54^.

### Participants

The participants of the presented study were employees of Generation Z from Poland. Generation Z consists of young people born between 1995 and 2004.

In the first stage of the study, before Covid-19, sample size was 200, but in the second stage, during Covid-19, 102 participants were qualified. As some research results indicate^28,41,49^ not too big sample size (i.e. between 10 and 50) not a large sample size (i.e. between 10 and 50) is needed in order to examine and assess it using factor analysis, as long as communalities are high, the number of expected factors is relatively small, and the model error is low. Based on the mentioned studies, we are certain that the data met the quality criteria necessary to perform the factorial analysis^52^. A factorial analysis of 8 Likert scale questions from this attitude survey was conducted on data gathered from 200 participants from Poland.

The “generational” approach taken by us in the paper discuss the conceptual nature of generations more clearly. We are aware, that some researchers pay attention on how to differentiate generational effects from age or cohort effects^13,46^. However, viewing the employees from the point of generational perspective is of great importance, though not the most crucial factor in providing contextual influences on employee motivation. Conceptual nature of generations should pay some attention on how to differentiate generational effects from age or cohort effects. In order to determine whether a cohort effect is present, we plan a cohort study in the future.

### Ethics statement

All participants provided informed consent and were informed about the possibility of quitting the survey anytime without consequences. The survey was conducted following the 1964 Declaration of Helsinki with later amendments. Their personal anonymity was preserved (although the relevant data are known to the authors). The survey was distributed by the various Internet Platforms used the quatrics^55^ to send the proprietary questionnaire.

The study involves no ethical concerns, and the study materials and design have been approved by the Institutional Review Board (Ethics Committee) WSB University in Toruń, Poland-the approval was issued on 20.20.2019. All methods were performed in accordance with the relevant guidelines and regulations contained in Editorial and publishing policies.

## Results

### Reliability and validity

The research problem described in previous sections was investigated using the factor analysis with Equimax rotation. For the part Q3 of the questionnaire containing together 8 items with the grades from 0 to 5, we obtained factors extracted in the Factorial Analysis. The reliability of the Q3 part of the questionnaire is confirmed by computing the Cronbach’s alpha and McDonald’s total omega.

The values are 0.70 and 0.73, before and during Covid-19, respectively, showing acceptable internal reliability for early-stage research^18,47,64^. However, it is worth to notice, that there is considerable discussion of misconceptions about the proper choice of cut-off level of Cronbach’s alpha value (for review, see e.g.^35,47,64,68^). Many studies assume that 0.8 is the proper value of cut-off, but many preliminary and validation research assume Cronbach’s alpha value threshold 0.7^5,24,47^.

One of the way to avoid above mentioned misunderstand is the redesign of the research. In our case this possibility is not applicable because Covid-19 is the unexpected occurrence situation. Further looked at post-modifying the data by removing items of the questionnaire to increase already acceptable Cronbach’s alpha was also considered. Nevertheless, the possible issues, e.g. unnatural inflation of the coefficient^32^, modification of the data unsupported by theoretical motivation, states against this option.

Taking into account mentioned questions and following modern research, more accurate comparative reliability measures are used like McDonald’s omega. This coefficient better estimates reliability for multifactor data, and Cronbach’s alpha underestimate it ?,^69^.

The related McDonald’s total omega is 0.78 for the data during the Covid-19 pandemic and 0.79 for the data before the pandemic. The threshold for scientific acceptable values (0.8) is the same as for Cronbach’s alpha.

### Factor analysis results for Q3 before Covid-19

In this part of the analysis, we examined Generation Z’s expectations towards a new approach to motivating companies. According to the scree test criterion and Kaiser method, the Q3 data generated three factors with eigenvalues greater than one, corresponding to 65.3% of the variance explained. Factor 1 (F1) with 31.7% explaining the most variance from other factors, factor 2 (F2) with 16.95% of the variance, and factor 3 (F3) with 16.64% of variance. The Equimax rotation method has been used. In Table 1 a rotated factor matrix is presented.

**Figure 1:**
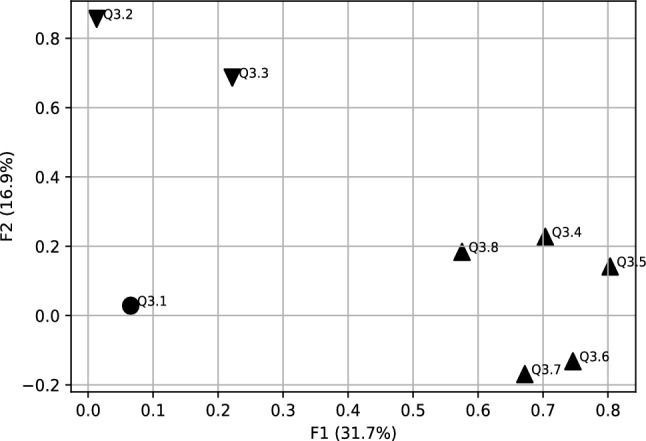
2D factors plot for Q3 data before Covid-19 (correlation matrix FA).

**Figure 2:**
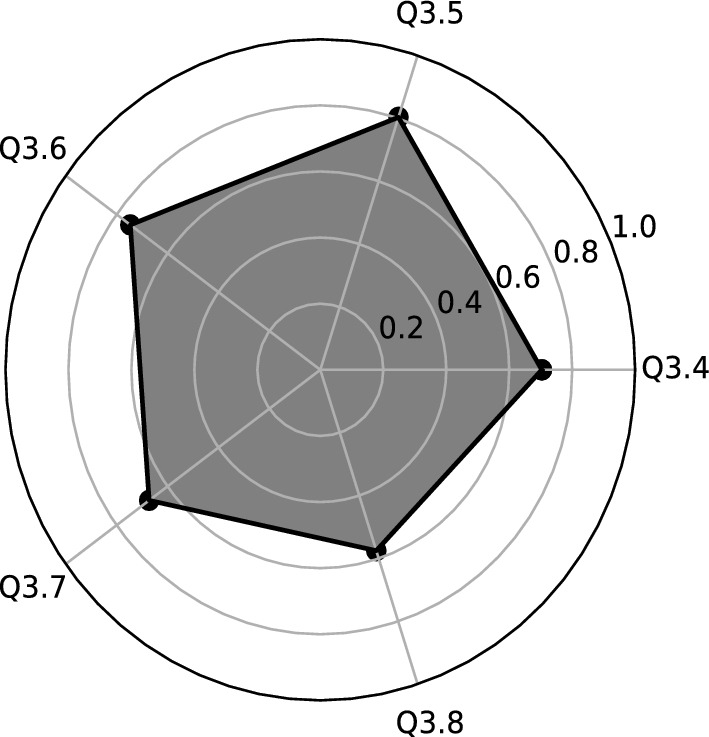
Graphical interpretation of the first factor (F1) of Q3 before Covid-19: Hygge Star.

Variables with higher correlation have been selected for each new factor (see Table 1 and Fig. 1):

- **F1** (the first factor ) is strongly correlated with the following variables of *the concept of “hygge”*: Q3.4–Q3.8. It is clearly visible that these variables created the five-arm star (Fig. 2). We call it *Hygge star.* Every arm of star corresponds to different area of hygge concept.
- **F2** (the second factor) has the variables Q3.2–*Cafeteria system* with a correlation 0.86 and Q3.3–*Flexible remuneration system* with correlation 0.7. This is the second important factor, with an explained variance of 16.95% Factor 2 can be called *a flexible remuneration system based on the cafeteria systems.*
- **F3** (the third factor) is strongly correlated with the variable Q 3.1–*Work-life balance concept* (with 0.83 correlation), therefore we can define it as *a balance between personal and professional sphere.* This is the third most important factor, with an explained variance of 16.64%

**Table 1:** Rotated factor matrix for Q3 before Covid-19.

Variables	Factors		
	F1	F2	F3
Q3.1: Work-life balance concept (keeping balance between your private and professional life)			0.83
Q3.2: Cafeteria system (possibility of choosing your own benefits from a list offered by the employer)		0.86	
Q3.3: Flexible remuneration system (wages are adjusted to the employee’s competencies and results)		0.7	
Q3.4: Concept of “hygge” in the area of designed office space with plants and eco-friendly elements	0.8		
Q3.5: Concept of “hygge” in the area of flat organizational structure, egalitarianism, and transparency at workplace	0.80		
Q3.6: Concept of “hygge” in the area fair play and includes not taking aggressive actions on the business market	0.75		
Q3.7: Concept of “hygge” in the area of organizational culture which includes respect towards one another, teamwork, integration and communication	0.67		
Q3.8: Concept of “hygge” in the area of role of the manager-leader, who positively motivates the employees, is available to everyone, and is part of the team	0.58

### Factor analysis results for Q3 during Covid-19

Now, we present the results concerning Generation Z’s expectations towards a new approach how to motivate young people during Covid-19. The scree test criterion and Kaiser method, indicate that the Q3 data generated three factors with eigenvalues greater than one, corresponding to 61.1% of the variance explained. Factor 1 (F1) with 24.1% explaining the most variance from other factors, factor 2 (F2) with 22.3% of the variance, and factor 3 (F3) with 14.7% of variance. We applied the Equimax rotation method as well. The Table 2 presents a rotated factor matrix.

**Figure 3:**
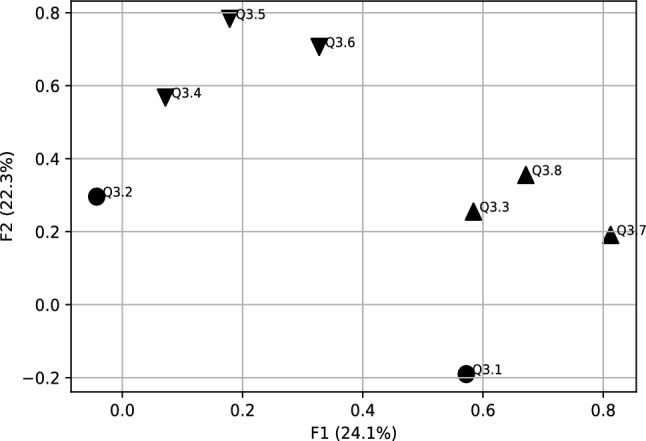
2D factors plot for Q3 data during Covid-19 (correlation matrix FA).

**Figure 4:**
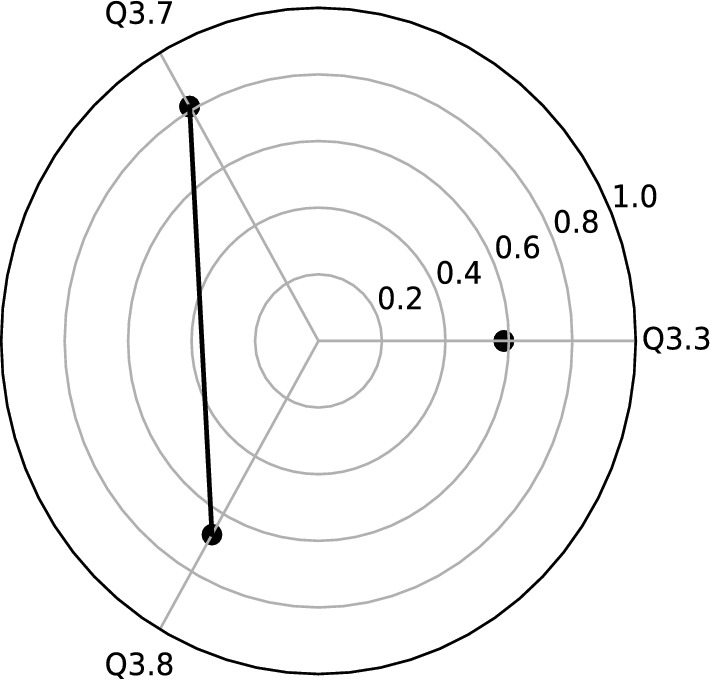
Graphical interpretation of the first factor (F1) of Q3 during Covid-19: Hygge segment.

**Figure 5:**
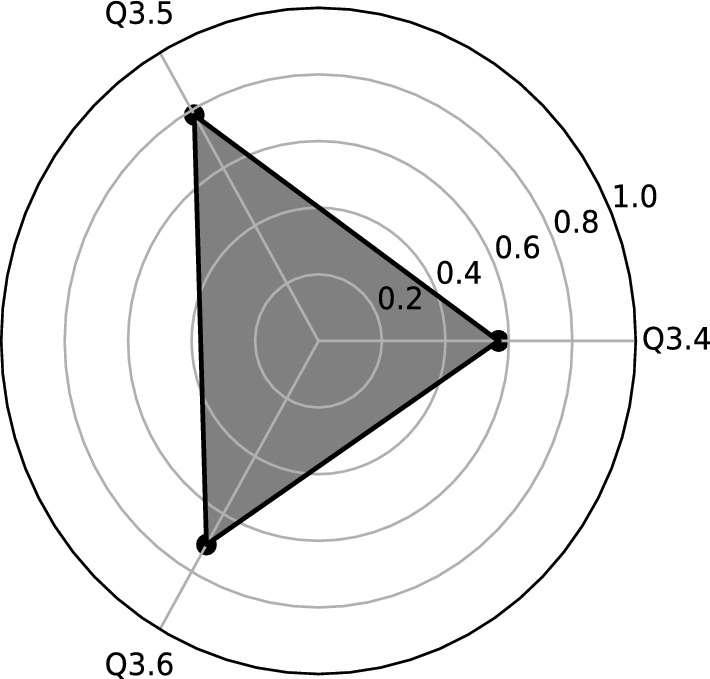
Graphical interpretation of the second factor (F2) of Q3 during Covid-19: Hygge pyramid.

Variables with higher correlation have been selected for each new factor (see Table 2 and Fig. 3):

- **F1** (the first factor ) is strongly correlated with two variables of *the concept of “hygge”*: Q3.7, Q3.8 and Q3.3-*Flexible remuneration system*. It is clearly visible that Hygge variables can create the segment, so we call this factoe *Hygge segment* (Fig. 4).
- **F2** (the second factor) has the three variables of *the concept of “hygge”*, that are Q3.4–Q3.6. This important factor, with an explained variance of 22.3% can be called *Hygge pyramid.* The Fig. 5 presents geometrical interpretation of *Hygge pyramid.*
- **F3** (the third factor) is strongly correlated with the variable Q 3.1–*Work-life balance concept* (with 0.61 correlation) and Q3.2–*Cafeteria system* (with 0.83 correlation). This factor explained 14.7% of total explained variance.

**Table 2:** Rotated factor matrix for Q3 during Covid-19.

Variables	Factors		
	F1	F2	F3
Q3.1: Work-life balance concept (keeping balance between your private and professional life)			0.61
Q3.2: Cafeteria system (possibility of choosing your own benefits from a list offered by the employer)			0.83
Q3.3: Flexible remuneration system (wages are adjusted to the employee’s competencies and results)	0.58		
Q3.4: Concept of “hygge” in the area of designed office space with plants and eco-friendly elements		0.57	
Q3.5: Concept of “hygge” in the area of flat organizational structure, egalitarianism, and transparency at workplace		0.78	
Q3.6: Concept of “hygge” in the area fair play and includes not taking aggressive actions on the business market		0.71	
Q3.7: Concept of “hygge” in the area of organizational culture which includes respect towards one another, teamwork, integration and communication	0.81		
Q3.8: Concept of “hygge” in the area of role of the manager-leader, who positively motivates the employees, is available to everyone, and is part of the team	0.67

## Discussion

We verified stated hypothesis H1 positively:

### H1

The Hygge star model dimensions to employees’ overall work motivation are valid but not invariant across surviving interminable disruptions (e.g., the corona virus COVID-19 pandemic).

The present research developed a new measure of work motivation based on the Hygge star. We tested for the reliability, factorial structure, and validity of this new scale before and after the pandemic time.

Young workers, in particular, demonstrate high importance regarding the balance between private and professional spheres. For Generation Z, high motivation to work is synonymous with achieving a harmonious and satisfying life in all areas of functioning. They need more time to care for their loved ones and expect to leave the working day in a crisis. The development of work-life balance is influenced by the need for self-fulfillment, which can be achieved thanks to a satisfactory professional situation and a successful private life.

The expectations of Generation Z employees before Covid-19 pandemics in terms of motivation focus on balance between all areas of the Hygge concept. If all areas (star arms) are applicable in an enterprise, there is a high probability that employee motivation will be high or very high. On the other hand, when there is a situation where hygge areas occur to a small extent or not at all, it can be assumed that employees will display a much lower level of employee motivation.

The above assumptions of *the Hygge star model* gave grounds that the Authors perceive the Hygge concept as a *new tool* for measuring the degree of motivation of Generation Z employees at the workplace. In our approach, we assume that the greatest motivation of Generation Z employees occurs when the company has identified all five hygge areas arranged in the structure of a five-pointed, regular star (pentagram). Thus, the ideal situation is when the Hygge star arises as a regular pentagon (the intersection of all diagonals of the regular pentagon). In that case, a balance is maintained between all the hygge areas, as expected by Generation Z employees.

Our new concept of approach to motivation of Generation Z employees is based on empirical studies of young people’s expectations, according to which the hygge concept should include all arms (areas) of the Hygge star. If all areas (star arms) are present in the structure of the motivation model of a given enterprise, there is a high probability that employee motivation will be at a high or very high level. On the other hand, in the case of little or no hygge areas, we assume that Generation Z employees will have a much lower level of motivation.

**Figure 6:**
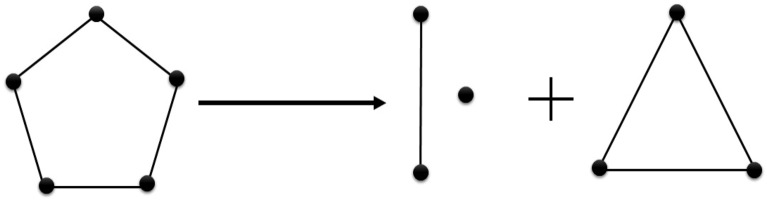
The intuitive visualization of the split of the Hygge star (pentagon) during COVID-19 pandemics into simplices (point, segment and triangle). See description in the text.

On the other hand, due to changing the work habits to more remote ones because of pandemics, the Hygge star splits into *a segment, a point and a triangle* that vividly indicates the paradigm shift in the concept of motivation. It can understand vaguely as *a simplex*^26^, point, segment triangle), used in algebraic topology, that are base elements of the pentagram that appeared before the pandemics (cf. Fig. 6).

We proposed the *Hygge pyraimid* as a new approach of motivation that arose during the pandemic time. It consists of three Hygge components (questions) related to the eco-friendly office design, transparency at the workplace, egalitarianism, and fair play actions. Moreover, for young people, a company with non-aggressive marketing actions is vital to similar concepts.

Another geometric structure we distinguish during pandemic times was named the *Hygge segment* (and a point). The concept is related to a flexible remuneration system, which means that the motivational tools are attached to the competencies of employees and the results of their works. The proposed segment consists only two components (questions) related to Hygge concept: organization culture with teamwork, integration, and communication, and the specific role of manager/leader, who positively motivates the employees and is part of the team. This hidden structure strongly suggests that this approach can ensure high motivation, which can translate to the success of the company. In the future, we plan to extensively investigate such hidden structure interconnections within the Hygge concept.

### Strengths and limitations

This research has several strengths, complex sampling, data gathered to validate the Hygge star scale before and after the pandemic time, which show the changes, which Covid-19 really made. Limitation of our study is the reliance upon a single country. For future research in this field it would be of great help to examine our focal theme from numerous alternative geographical settings^33^. The next step is cross-culturally validated scale. It will allow us to conduct stringent research on cross-cultural similarities and differences in motivational processes in work environments.

## Implications

At the level of organization it is vital that relevant practitioners such as HR managers note that Generation Z employees:

- require new solutions in the area of motivation;
- are only motivated when there is a good atmosphere in the company, including good relations with the boss and colleagues;
- treat the company as an authority when they receive a lot of support from their boss and are convinced that they have a bright future in this organization;
- are satisfied and the level of their commitment increases when companies implement modern motivational concepts, e.g., hygge;
- should implement the Hygge star model, the so-called “Work Happiness Model”.

We tried to give specific recommendations on concrete steps that employers could take to improve the motivation of young workers.

According to Authors Hygge star model, the so-called “Work Happiness Model” should consist of the following components:

- Workplace: Cosy, interestingly designed office space and the presence of greenery with eco-friendly elements;
- Organisational Structure: Flat organisational structure, egalitarianism and transparency at the workplace;
- Activities in Business (social responsibility): Fair play and lack of aggressive business actions on the market;
- Organisational Culture: Culture includes mutual respect, a culture of teamwork, integration and communication, as well as the key role of the manager or the leader;
- Motivating Employees: Engagement of employees based on good relations, clarity of goals and rules, proper work evaluation and feedback from the employer/manager;

Hygge can be effective in enterprises when we apply it in the area of motivation, reorganize the organization of work in the company according to hygge, and restructure the hierarchy and make changes in business activities. Enterprises which are implementing hygge can also be recognized by introducing greenery into the workplace. Plants relax and calm employees who are performing their duties. Hygge opts for egalitarianism and transparency, which has a positive effect on the equal treatment of employees and the transparency of motivation processes and methods of remuneration. There is no hygge without fair play and excluding aggression from the workplace.

We advise that the main step for companies is to implement the concept of hygge (happiness at work) which emphasizes the role of the manager-a leader who positively motivates employees and who is accessible to all and is part of the team. Our findings in this regard confirm previous research^20,37,43–45^.

We also infer from our research the changes that occurred about the area of “Work Happiness Model” of Generation Z during the Pandemic, which is a new contribution. With the help of our research, we have created the Hygge pyramid as a new approach to motivation that has emerged during the pandemic. It consists of three Hygge components: egalitarianism and fair play activities related to green office design, transparency in the workplace and non-aggressive marketing activities. For companies with little experience in building happiness-based motivation, we recommend via: Frandsen, Johansen^21^, and Jasielska^29^: to take care of employee happiness, implement a Chief Happiness Officer (CHO) position. This relatively new position is gaining enthusiasts around the world: CHOs are employed by Google and the government of the United Arab Emirates, for example. There are many interpretations and conceptions of what the designated CHO will do^48^. The term is used by consultants who offer advisory services to implement new solutions (for example, the Danish company WooHoo). They can, for example, offer a series of positive leadership training courses for managers in the company, or offer ready-to-implement solutions based on their best business practices and analysis of the situation in the organization^29,65^ or be advisors on how to create organisation models for a friendly and effective communication to foster happiness at work.

At the level of HE institutions, it is important that practitioners such as careers counselors encourage graduates to develop teamwork and brainstorming. Employability programs in HE institutions should focus on helping students find the appropriate company that operates according to the concept of *hygge* based on mutual trust, teamwork, transparency of actions and decisions, as well as chill, liberty, and spontaneity. Practitioners also should notice that hygge in the workplace develops teamwork and brainstorming. Employees look for solutions together. The structure in such an enterprise is flat, which directly improves communication and reduces the distance between colleagues. Our study indicates that, in addition, further development of the hygge concept may be required to develop happiness at work.

## Conclusions

As businesses and organizations are globalizing, researchers and practitioners must find ways to help managers and organizations engage diverse employees. To accomplish this, they must understand how this can be achieved. Finally, the authors suggest to use motivators that are valued by each of the three generations, i.e. X, Y and Z, before being able to attract the best candidates of each generation. Organizations should not only create an inclusive and understanding multigenerational working environment but also be able to communicate strong branding via new communication channels successfully (e.g., social media networks), which Generation Z utilise better than any other generation in employment. We suggest that organisations with diverse generational composition should adopt new instrument of motivation and use more elastic instruments in the motivation level to survive interminable disruptions e.g., the corona virus COVID-19 pandemic. We found that there is a growing need for a scale like and model of the Hygge star to foster future research that will continue to accurately assess relations between the different types of motivation and organizationally relevant variables

### Supplementary Information

Supplementary Information.

## Data Availability

The datasets used and/or analyzed during the current study are available from the corresponding author on reasonable request. All data generated during this study are included in this article.
