# Supplementary material for: Structured multi-criteria model of self-managed motivation in organizations based on happiness at work: pandemic related study
Source: Sci Rep. 2023 Oct 2;13:16521. doi: 10.1038/s41598-023-43626-5 (PMC10545680; doi:10.1038/s41598-023-43626-5)
Supplement: Supplementary file 1 — Supplementary Information. [file 41598_2023_43626_MOESM1_ESM.pdf]

Supplementary information. Questionnaire

Dear Participants,
I would like to invite you to participate in research devoted to selected issues in managing generational diversity. The goal of the research is to identify effective tools for motivating of employees from the perspective of representatives of Generation Z. When you are answering the questions in the survey, please select only one answer on the given measuring scale. If you don't know the answer to a particular questions, just please leave it blank. The questionnaire is anonymous. The results will only be used for scientific research. I'd like to thank you very much for participating in the research.

Directions: Please indicate your answer by tick the appropriate number. The higher level the more important the modern systems and concepts of remuneration and motivation.

(Note: 1 → unimportant 2 → not so important 3 → moderately important 4 → important 5 → very important)

Q3: *How important are the following modern systems and concepts of remuneration and motivation for you?*

	1	2	3	4	5
Q3.1. Work-life balance concept (keeping balance between your private and professional life)	☐	☐	☐	☐	☐
Q3.2. Cafeteria system (possibility of choosing your own benefits from a list offered by the employer)	☐	☐	☐	☐	☐
Q3.3.Flexible remuneration system (wages are adjusted to the employee's competencies and results)	☐	☐	☐	☐	☐
Q3.4.Concept of "hygge" in the area of designed office space with plants and eco-friendly elements	☐	☐	☐	☐	☐
Q3.5.Concept of "hygge" in the area of flat organizational structure, egalitarianism, and transparency at workplace	☐	☐	☐	☐	☐
Q3.6.Concept of "hygge" in the area fair play and includes not taking aggressive actions on the business market	☐	☐	☐	☐	☐
Q3.7.Concept of "hygge" in the area of organizational culture which includes respect towards one another, teamwork, integration and communication	☐	☐	☐	☐	☐
Q3.8.Concept of "hygge" in the area of role of the manager – leader, who positively motivates the employees, is available to everyone, and is part of the team	☐	☐	☐	☐	☐
